# Insights into the Genetic History of French Cattle from Dense SNP Data on 47 Worldwide Breeds

**DOI:** 10.1371/journal.pone.0013038

**Published:** 2010-09-30

**Authors:** Mathieu Gautier, Denis Laloë, Katayoun Moazami-Goudarzi

**Affiliations:** 1 INRA, UMR 1031 CBGP, Montferrier-sur-Lez, France; 2 INRA, UMR 1313 GABI, Jouy-en-Josas, France; Innsbruck Medical University, Austria

## Abstract

**Background:**

Modern cattle originate from populations of the wild extinct aurochs through a few domestication events which occurred about 8,000 years ago. Newly domesticated populations subsequently spread worldwide following breeder migration routes. The resulting complex historical origins associated with both natural and artificial selection have led to the differentiation of numerous different cattle breeds displaying a broad phenotypic variety over a short period of time.

**Methodology/Principal Findings:**

This study gives a detailed assessment of cattle genetic diversity based on 1,121 individuals sampled in 47 populations from different parts of the world (with a special focus on French cattle) genotyped for 44,706 autosomal SNPs. The analyzed data set consisted of new genotypes for 296 individuals representing 14 French cattle breeds which were combined to those available from three previously published studies. After characterizing SNP polymorphism in the different populations, we performed a detailed analysis of genetic structure at both the individual and population levels. We further searched for spatial patterns of genetic diversity among 23 European populations, most of them being of French origin, under the recently developed spatial Principal Component analysis framework.

**Conclusions/Significance:**

Overall, such high throughput genotyping data confirmed a clear partitioning of the cattle genetic diversity into distinct breeds. In addition, patterns of differentiation among the three main groups of populations—the African taurine, the European taurine and zebus—may provide some additional support for three distinct domestication centres. Finally, among the European cattle breeds investigated, spatial patterns of genetic diversity were found in good agreement with the two main migration routes towards France, initially postulated based on archeological evidence.

## Introduction

As part of the Neolithic agricultural revolution, the domestication of cattle, which occurred about 8,000 years ago, changed the social and economical life of most human populations [1] and contributed to the gradual transition of hunter gatherers into farmers with permanent settlements. Although fully interfertile, we distinguish two taxa of domestic cattle - humpless taurines (*Bos taurus*) and humped zebuines (*Bos indicus*) [2]. Archeological evidence mostly osteometric and morphometric data early argued in favor of several separate cattle domestication events [3] which was supported by more recent genetic analyses based on mitochondrial DNA [4], [5] and Y-chromosome polymorphisms [6]. However the number of domestication centres remains a source of intense debate and disagreement. Two main hypotheses have been formulated [2]: (i) two domestication events: a first major domestication event of taurine cattle in the Fertile Crescent (*i.e.* between the Mediterranean sea and Iran) from the wild extinct aurochs *Bos primigenius primigenius* and a second separate one which lead to zebus in the Indus valley (including Rajasthan and present day Pakistan) from the wild extinct aurochs *B. p. namadicus* [7] and (ii) a total of three domestication events: the two previous ones and a third one leading to African taurines in Northeastern Africa from the wild extinct aurochs *B. p. opisthonomous* [3], [8]. During the 3,000–4,000 years after domestication, cattle expansion had followed different and complex routes tightly related to the migration of early breeder populations and the spread of agriculture over Europe, Africa and Asia [3]. Nevertheless, the overall diffusion of populations was estimated to take place at a slow and continuous rate of around 1.1 km per year [9]. Throughout Europe, early breeders presumably spread from the Fertile Crescent towards North-West following two distinct routes [3]. One group of farmers progressed to the North along the Balkans' rivers (following the so-called Danubian route) establishing the Neolithic culture in Germany and the Netherlands approximately 6,500 BP. A second group migrated to the West via maritime routes across the Mediterranean Sea (following the so-called Mediterranean route) establishing the Neolithic culture in Italy 6,500 BP or in Spain and France 6,000 BP [3]. Nevertheless, a number of secondary livestock migrations might have accompanied human migrations in more recent historical times. Similarly, during these migration waves, some sporadic events of interbreeding between wild European aurochs (*B. p. primigenius*), which had been present until the Middle Age, and domestic stocks might have occurred to a substantial extent [2], [4], [10]. These complex origins of cattle associated with both natural and artificial selection gave rise to numerous different breeds displaying a broad phenotypic variety over a short period of time. In France, it is generally believed that some aspects of the Neolithic culture originated from central Europe and also via the Mediterranean route. A fine scale characterization of the genetic structure of French cattle breeds might thus be expected to display footprints of such migrations and provide in turn additional insights into the establishment of the Neolothic culture.

The recent advent of high-throughput and cost effective genotyping techniques makes it possible to provide a detailed genome wide assessment of the genetic structure and relationships among cattle populations. This might in turn allow to refine previous pioneering works usually performed on a small number of genetic markers (*e.g.* [11], [12]). We present in this study a detailed analysis of cattle diversity based on 1,121 individuals sampled in 47 populations from different parts of the world (with a special focus on French cattle) genotyped for 44,706 autosomal SNPs. More precisely, the data set consisted of new genotypes for 296 individuals representing 14 French cattle breeds which were combined to those available in three previous published studies: i) 19 populations sampled by the Bovine Hapmap Consortium [13] and genotyped with the Illumina® BovineSNP50 chip assay [14], ii) 11 African populations [15] and iii) 3 French dairy cattle breeds described in [16]. After characterizing SNP polymorphism in the different populations, we performed a detailed analysis of genetic structure at both the individual and population levels. This confirmed a clear partitioning of cattle diversity into distinct breeds. In addition, the overall pattern of differentiation among three main groups of populations (African taurine, European taurine and zebuine cattle) may provide some additional support for three distinct domestication centres. We further searched for spatial patterns of genetic diversity among 23 European populations, most of them being of French origin under the recently developed spatial Principal Component analysis (sPCA) framework [17].

## Results and Discussion

### SNP data, polymorphism and Linkage Disequilibrium

We first performed a joint analysis based on SNP data generated for all 1,121 individuals representing 47 different populations (24 individuals per population on average) genotyped for 44,706 SNPs from this study and three previously published studies (see Materials and Methods and Table S1) to provide a global picture of cattle genetic diversity. As detailed in Table S1 and in agreement with previous studies [13]–[15], SNP average heterozygosity was found higher in populations from European origin (from 0.2544 for JE2 to 0.3156 for PRP) compared to zebu cattle (from 0.1556 for GIR to 0.1945 for ZMA) and taurines from West Africa (from 0.1828 for LAG to 0.2240 for SOM) or East Africa (0.2432 for SHK). As previously discussed [14], [15], this trend might be directly related to the ascertainment bias introduced in the construction of the BovineSNP50 chip assay, SNPs being almost exclusively derived from sequences available in European cattle breeds. Using such data to infer genetic divergence among cattle might thus be done cautiously and is expected to bias the estimation of genetic divergence between more distantly related populations (*e.g.* European and African taurines or zebus). Interestingly, populations of hybrid origin displayed generally higher levels of polymorphism than their population of origin as exemplified for i) the two synthetic breeds, BMA and SGT, which result from crosses between European taurines and zebus [13]; ii) the West African hybrids (BOR and KUR) and to a lower extent West African zebus (ZBO and ZFU) which result from crosses between West African taurines and zebus and iii) the Moroccan breed OUL which has a probable hybrid origin between European and African taurine (see below).

As shown in Figure S1 and previously reported (*e.g.* [18]), the average within-population pairwise *r^2^* dropped quickly toward its asymptotic value when physical distances were above 200 kb. In our data set, SNP genome coverage was homogeneous with a very small proportion of inter-marker distances less than 20 kb (Figure S2, Table S2 and Materials and Methods) and with 3.5 SNPs (from 0 to 9) per 200 kb on average. Thus most SNP pairs in this study displayed a level of within population Linkage Disequilibrium (LD) close to that observed between unlinked SNPs. We thus did not consider in the following any spatial dependencies among SNPs which might result from LD and subsequently carried out descriptive analyses to further assess the structure of genetic variability at both the individual (ignoring the information on the population of origin) and population levels.

### Assessing the genetic structure at the individual level

**Figure 1:**
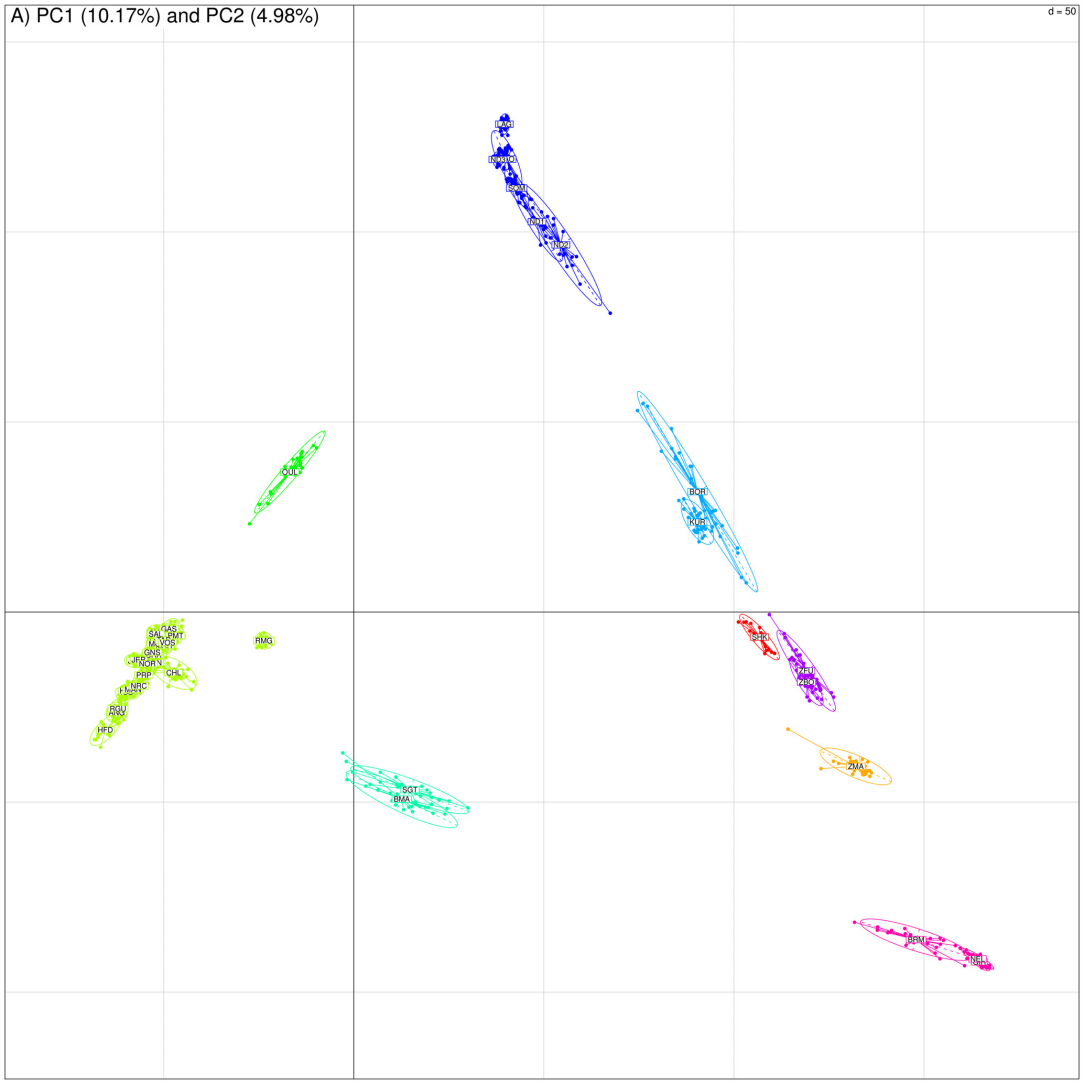
PCA results obtained with the whole data set (1,121 individuals, 44,706 SNPs). Individuals are plotted according to their coordinates on the first two principal components. Ellipses characterize the dispersion of each breed around its center of gravity (assuming the cloud is a random sample distributed according to a bivariate gaussian distribution, the probability for an individual to be within the ellipse is 0.9).

We first carried out a principal component analysis (PCA) based on all available SNP information allowing to refine and extend previous reports [13], [15]. In particular, from a worldwide perspective, the data from [14] and [15] were very complementary since the 19 Hapmap populations [14] (also analyzed with 37,470 SNPs in [13]) contained only two populations from Africa (ND3 from Western Africa and SHK from Eastern Africa). On the other hand, 10 African populations (eight from Western Africa, one from Northern Africa and one from Madagascar) were surveyed by [15] but (pure) zebuine populations were lacking in this data set. As shown in Figure 1, the first component which accounted for 10.17% of variation resulted in the separation of the underlying populations according to a zebuine/taurine gradient while the second one (accounting for 4.98%) could be interpreted as a European/African taurine gradient. The resulting 2-Dimensional global organization of cattle genetic diversity might thus be described as a triangle with apexes corresponding respectively to European taurines (EUT), West African taurines (WAT) and Zebus from Indian origin (ZEB). Following this representation, OUL individuals lay as expected on the EUT/WAT segment, BMA and SGT individuals on the EUT/ZEB segment (but closer to EUT) and West African hybrids (BOR and KUR) and zebus (ZBO and ZFU) on the WAT/ZEB segment. Similarly, SHK which is considered as a taurine population because humpless was positioned close to West African zebuine populations confirming previous reports [19].

**Figure 2:**
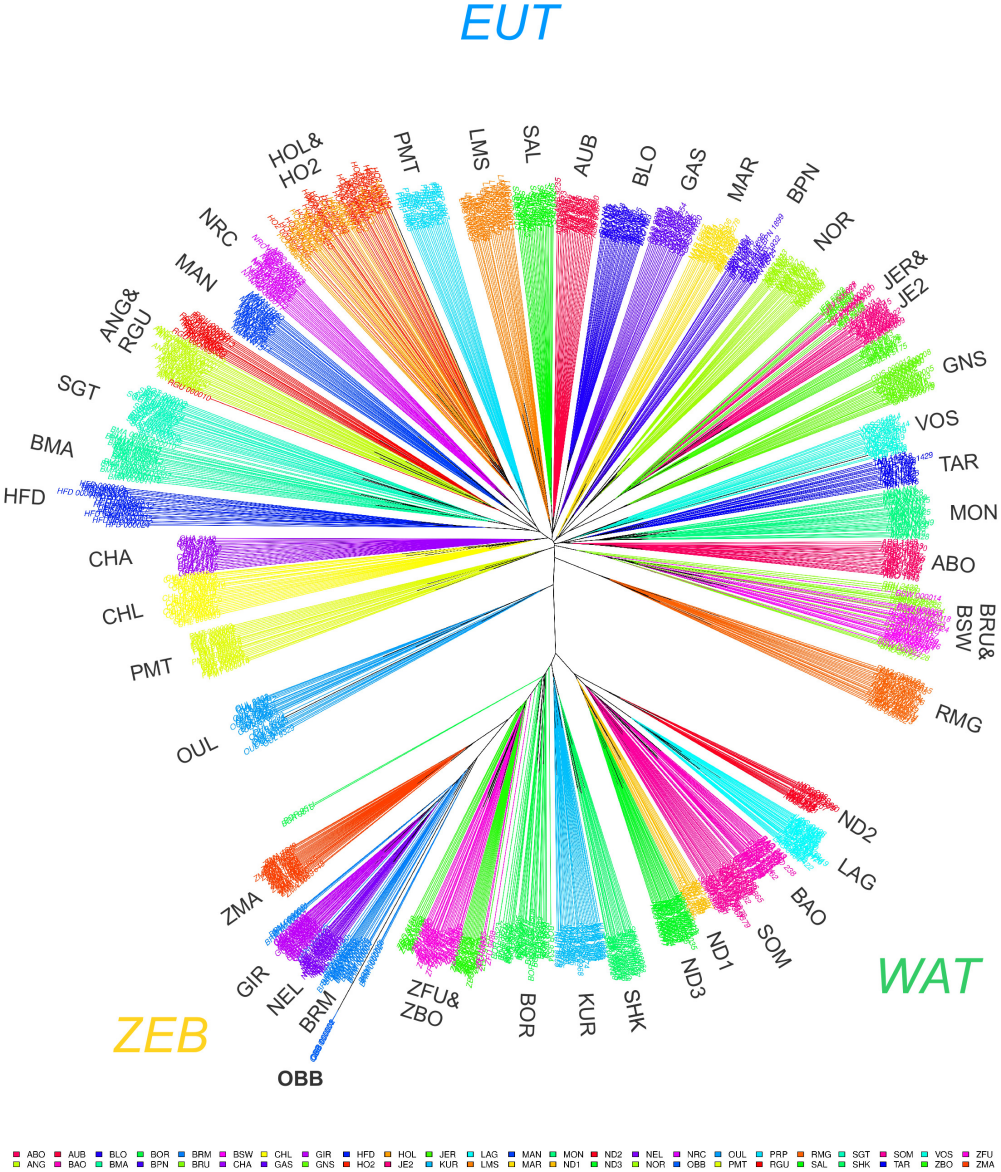
Neighbor-Joining tree relating the 1,125 individuals (1,121 cattle and 4 american bisons). The tree was constructed using allele sharing distances averaged over 44,706 SNPs. Edges are colored according to the individual breed of origin.

Besides, the neighbor-joining (NJ) tree based on Allele Sharing Distances (ASD) unambiguously separated individuals according to their population of origin (Figure 2) as confirmed when applying simple assignation tests (data not shown). As a consequence, at a higher hierarchical level, the three groups of populations corresponding to EUT, WAT and ZEB could also be clearly distinguished (respectively in upper, lower right and lower left Figure 2). In agreement with PCA results, individuals from West African hybrid populations (KUR, BOR) and West African zebus (ZFU and ZBO) branched in an intermediary position between WAT and ZEB; and OUL branched between EUT and WAT. Similarly, ZFU and ZBO were closer to ZMA and BMA and SGT branched within the EUT suggesting a lower influence of zebus than European taurines. In addition, among some of the closely related European cattle populations (similar breeds but different sample origin), BRU and BSW individuals, HOL and HO2 individuals and JER and JE2 individuals were almost indistinguishable suggesting that each of these different global populations might be considered as single populations as previously shown for the Holstein population [20]. However, a notable exception was observed for CHA and CHL individuals that were clearly separated.

**Figure 3:**
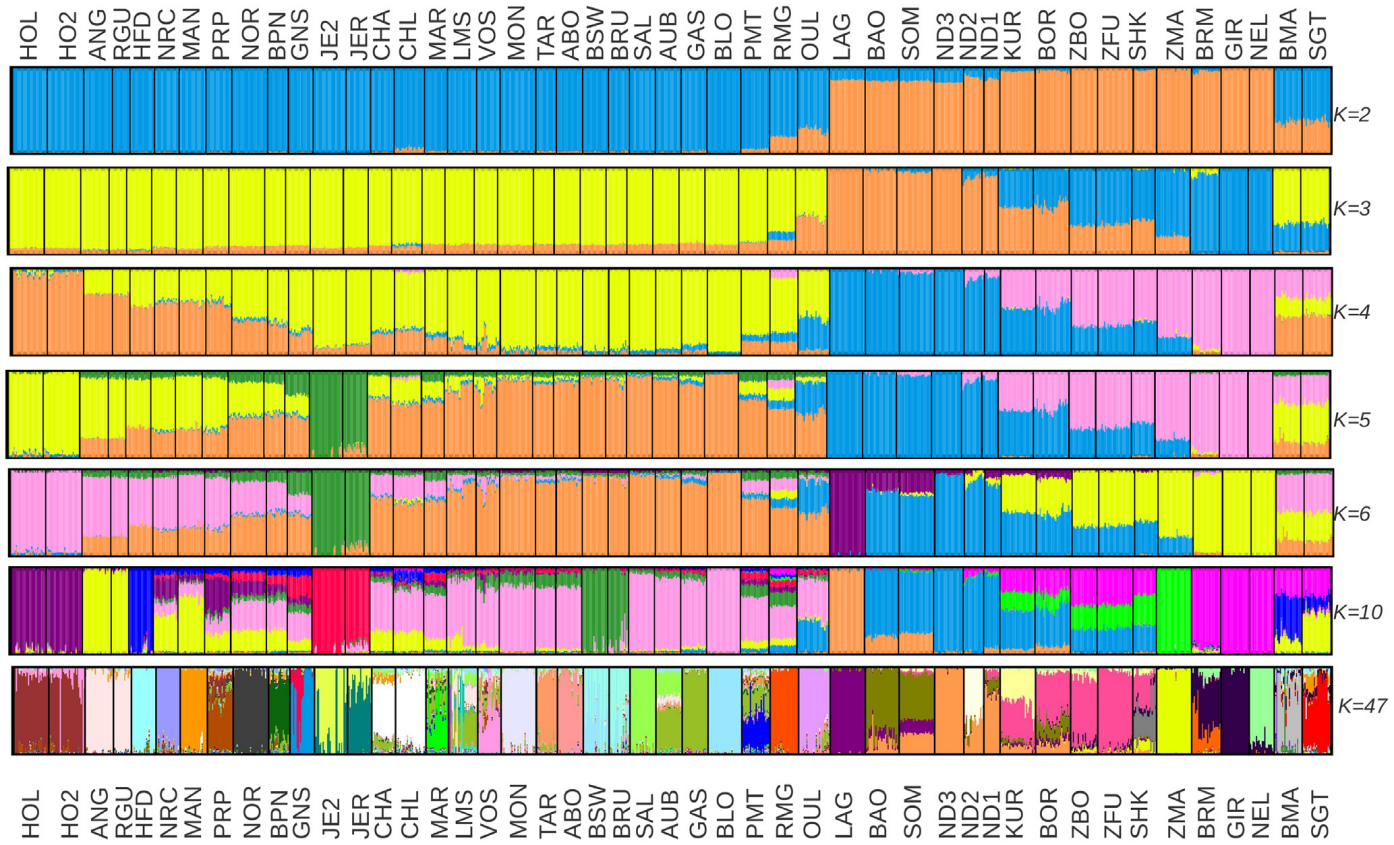
Unsupervised hierarchical clustering of the 1,121 individuals genotyped for 44,706 SNPs. Results for an inferred number of clusters K varying from 2 to 6, K = 10 and K = 47 (*i.e.* the number of breeds) are shown.

We finally performed a model-based unsupervised hierarchical clustering of the individuals using the program *frappe* [21]. As shown in Figure 3 (e.g. K = 3 and K = 4), results were in good agreement with above observations with a clear separation of EUT, WAT and ZEB. In addition, increasing the number of inferred clusters allowed to confirm the high admixture level and assumed origin of some populations (see above) such as West African hybrids (BOR and KUR) and zebus (ZBO and ZFU), SHK (which displayed similar characteristics as West African zebus [19]), OUL and synthetic breeds. Interestingly, among WAT, LAG (representative of shorthorn African taurines) individuals could be clearly (from K = 6) separated from ND3, ND2 and ND1 (representative of longhorn African taurines). These two later populations displayed a low level of zebu admixture not detected previously [15]. Nevertheless, SOM and BAO which are West African shorthorn taurines displayed a high longhorn influence. Similarly, the African taurine influence of OUL seemed to be of longhorn origin. Among ZEB populations, individuals belonging to ZMA separated (K>6) from those belonging to the other three ones (GIR, BRM and NEL) which were imported from India to Brazil (GIR and NEL) or USA (BRM) about 200 years ago [2]. The zebu genetic fraction of the West African hybrids (BOR and KUR) and noticeably SHK seemed to equally originate from these two zebus cluster (*e.g.* K = 10) while the infusion of zebus in SGT and BMA was of South American origin consistent with recent historical data [2]. Except for RMG and to a lower extent CHL (Charolais individuals sampled in the United Kingdom), EUT displayed no evidence of WAT or ZEB introgression (K = 6). The influence of WAT or ZEB in RMG (or other Italian breeds) has already been reported based on other kind of genetic data with i) the segregation of the T1 mtDNA haplogroup [10] or ii) the segregation of zebu associated microsatellite alleles [22].

Finally, individuals belonging to some breeds such as HOL (and HO2), ANG (and RGU), HFD, BSW (and BRU) tended to be clearly assigned to a single cluster when K = 10. This latter trend was further confirmed for most populations (although not all) when increasing the number of inferred clusters toward the number of populations (K = 47).

### Assessing the genetic structure at the population level

Overall, the different analyses performed at an individual scale suggested that the partitioning of cattle into distinct populations is relevant to characterize genetic diversity. This was expected, in particular when considering breeds originating from industrialized countries, and has already been reported for the 19 Hapmap populations [13]. Consistently, the *F_ST_* across populations was found equal to 0.190 with an average *F_IS_* almost null (−0.007) leading to an *F_IT_* of 0.185. Note that within all populations, *F_IS_* were also found close to zero (as close relationships among individuals were avoided during sampling) although moderately negative values (<−0.1) were observed for ND2 [15] and BLO (Table S3). *F_ST_* computed for each pair of populations (Table S4) ranged from 0.0044 (for HO2/HOL pair) to 0.4742 (for LAG/NEL pair) while within EUR populations, it ranged from 0.0044 (for HO2/HOL pair) to 0.2018 (for JE2/BLO pair).

We thus decided to extent the PCA described above by performing a so-called between-class PCA (*e.g.* [23]), classes being in our context identifiable to breeds. This latter analysis explicitly introduces population structure information in the PCA optimization criterion to find those axes that maximize the between-breed variance. Running both PCA and between-breed PCA allows under a model-free basis i) to compare the patterns of differentiation when performing the analyses at an individual level or at a population level and ii) to quantify the proportion of the total between-individual variance explained by the partitioning of genetic variability into breeds (between-breed variance). As shown in Figure S3, results from the between-breed PCA were highly similar to those obtained with the PCA on individual genotypes, the first three axes explaining more than 51.4% of the genetic variability across populations. Note that the third axis might be interpreted as North/South gradient among EUT (see below). Moreover, the correlations between the first twelve eigenvectors from PCA and between-breed PCA were almost equal to 1 (in absolute value). Thus, analyses at both the individual (PCA) and breed (between breed PCA) levels revealed highly similar patterns of population differentiation. Finally, comparing variances (*i.e.* sum of the eigenvalues) among the two analyses showed that genetic variability between populations explained 32.8% of the whole genetic variability (across individuals).

**Figure 4:**
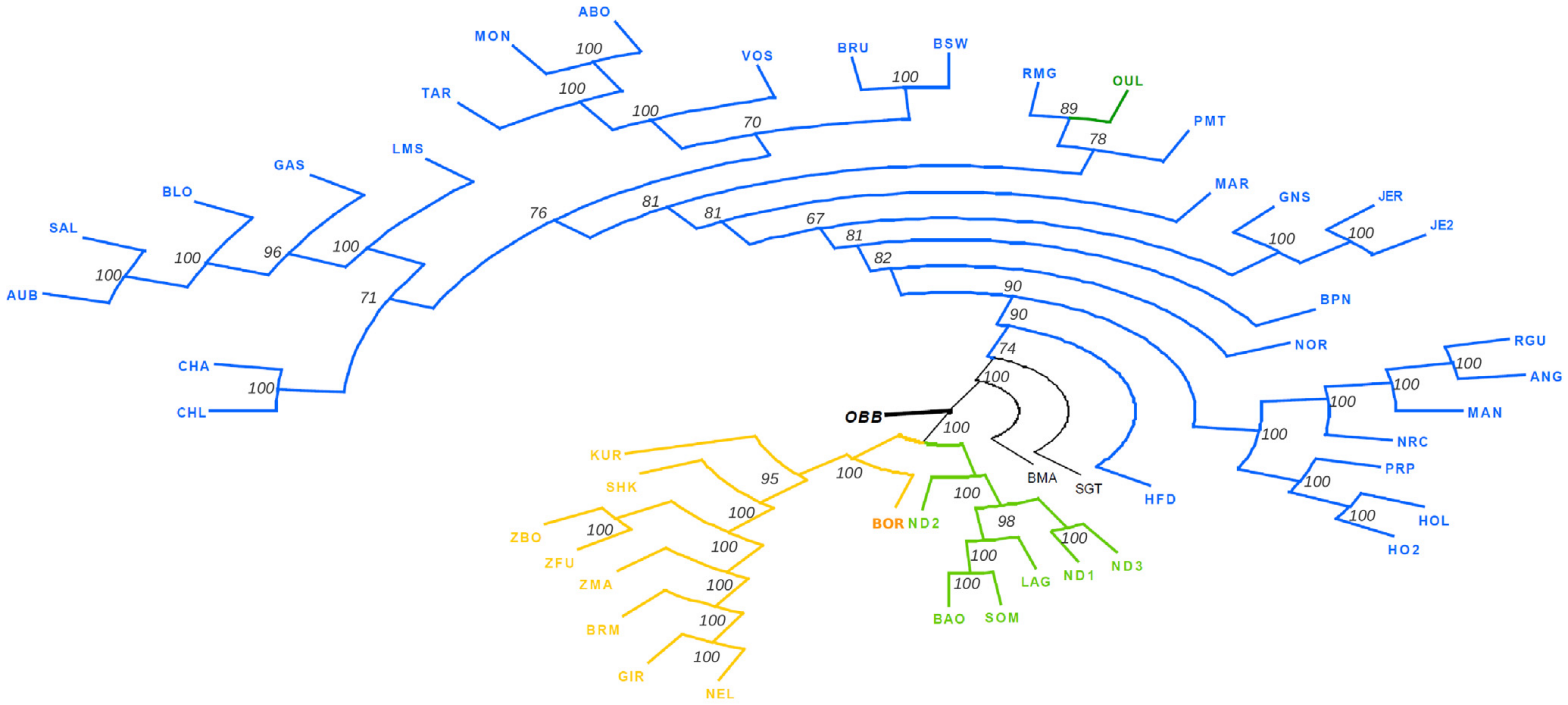
Neighbor-Joining tree relating the 47 cattle populations and American bison (OBB) outgroup based on Reynolds genetic distances computed using allele frequencies at 44,706 SNPs. Reliability of the nodes (percentage over 100 bootstrap samples) are indicated for each node.

We finally constructed a neighbor-joining (NJ) tree based on Reynolds genetic distances relating the 47 cattle populations and including American Bison (OBB) as a rooting outgroup (Figure 4). Given the amount of available information almost all nodes were highly reliable (node bootstrap values above 95%) and EUT (blue), WAT (green) and ZEB (orange) populations could be clearly separated. As expected, within WAT, longhorn taurines (ND1 and ND3) were separated from shorthorn taurines (BAO, SOM and LAG) although ND2, probably because of a higher zebu introgression (see Figure 3) branched immediately below the WAT node. Among African zebus and in agreement with above observations, ZMA was closer to zebus from Indian origin (NEL, GIR and BRM) than West African zebus (ZBO and ZFU) while SHK, BOR and KUR were in an intermediary position between WAT and ZEB. Likewise, SGT and BMA were in an intermediary position between ZEB and EUR although closer to EUR (see above).

Overall, these different analyses support a global partitioning of world-wide cattle diversity into EUT, WAT and ZEB. Although not excluding the possibility of strong founder effects, this might suggest three distinct domestication events [2]. This latter hypothesis is supported by genetic data based on mtDNA showing that the predominant haplogroup (namely T1) in Africa is absent in Europe and at a relatively low frequency in Anatolia and Middle East [10], [24]. Similarly, non recombining part of the cattle Y chromosome allowed the identification of three main haplogroups referred to as Y1 and Y2 for taurines and Y3 for zebus. Most haplotypes identified in African taurines are assigned to haplogroup Y2 and are not present in other continents [6], [25]. Interestingly, in the NJ tree (Figure 4), the OBB root clearly isolated EUT from the WAT/ZEB group which disagrees with the assumption of a common domestication center for EUT and WAT. Nevertheless, such a topology might be sensitive to the ascertainment bias favoring SNP from European origin. For instance, Figure S4 shows the NJ tree resulting from the analysis of a subset of 27,527 SNPs of an origin ancestral to the ZEB, WAT and EUT breeds separation. These were indeed chosen to display a MAF>0.01 in at least i) two breeds among ZMA, GIR, BRM and NEL ii) two breeds among ND1, ND3, SOM, BAO and LAG and iii) two breeds among 24 EUR. The positioning of the OBB root separated ZEB from the lower order group formed by WAT and EUR in agreement with the NJ tree based on ASD distances between individuals on the complete data set (Figure 2).

### A focus on the genetic structure of European breeds

Within EUR, grouping of the breeds on the NJ tree (Figure 4) was strikingly consistent with their geographical origin. Hence four main groups of breeds were found highly reliable (node bootstrap values equal to 100%) and lead to distinguish a) breeds from southwestern France (starting from the tips: AUB and SAL then BLO, GAS and LMS); b) breeds from Eastern French mountains (MON and ABO then TAR, VOS); c) breeds from the Channel Islands (JER and JE2 then GNS) and d) breeds from Northern European origin (ANG, RGU, HO2, HOL and NRC) together with the two French breeds MAN and PRP (see below). The Charolaise breeds (CHA and CHL) and the Brown Swiss breeds (BSW and BRU) branched with a less reliable node with groups a) and b) respectively. However, when considering tree resulting from the subset of 27,527 SNPs described above (Figure S4), they branched after the merging of groups a) and b). The resulting node joining a) and b) then displayed a higher bootstrap value (equal to 89%) than the node joining a) (and CHA and CHL) and b) (and BSW and BRU) on the tree of Figure 4 (node bootstrap value equal to 76%). Similarly in this latter tree, the two Italian breeds (RMG and PMT) and OUL branched with the group merging a) and b). Although OUL was also found close to RMG (relatively to the other EUR ones) in PCA (Figure 1), this positioning might be affected by ascertainment bias since these two breeds displayed substantial influence from African taurines and zebus respectively. Indeed and more consistently, in the tree of Figure S4, OUL and RMG were clearly separated from other EUR breeds. Subsequently, as shown in Figure 4, MAR (from northwestern France) was found at an intermediary position between the South European breeds (Italian breeds, groups a) and b)) and breeds from the Channel Islands (group c). This large resulting group finally branched with NOR and BPN (both originating northern than MAR) and group d). Finally, HFD was surprisingly the outgroup of other EUR breeds in Figure 4. Yet and more expectedly, HFD branched (node bootstrap value equal to 71%) with other North European breeds (constituted by groups c and d defined above with BPN and NOR) in the tree of Figure S4.

Overall, among European cattle, both NJ tree and PCA results suggested strong spatial patterns of genetic diversity. Hence, as pioneered by Cavalli-Sforza and collaborators for the reconstruction of the early history of human populations [26], interpreting the structure of such genetic structure in the light of geographical data is expected to provide insights into the underlying history of cattle [12]. However PCA does not take explicitly into account spatial information while grouping of populations based on the NJ tree is sensitive, to some extent, to ascertainment bias. We thus further searched for spatial patterns of genetic diversity under the recently developed sPCA framework [17], concentrating on French and other closer related European breeds.

### Spatial patterns of genetic diversity in French Cattle breeds

PCA does not explicitly incorporate geographical information because the optimization criterion relies on the maximization of the genetic variance. Thus, PCA may fail to detect spatial structuring if this is not associated with the most pronounced genetic differentiation. Recently, after the works of [27] and [28]; Jombart and collaborators [17] specifically developed a sPCA devoted to the analysis of allele frequency data, and showed that it performed better than PCA in retrieving simple spatial structures as well as more complex patterns among genotypes or populations. We thus used this approach to reveal the spatial patterns of genetic variation in French cattle breeds (in relation to other European ones) since they were particularly well represented in our data set. Hence, out of the 29 populations of European origin (Table S1), we only considered 23 breeds, discarding RGU (which derived from ANG), BRU (which is from the same origin as BSW), HO2 (which is from the same origin as HOL), JER (which is from the same origin as JE2), CHL (which derived from CHA) and RMG due to its zebu influence (see above). Geographical breed locations (Table S1) were summarized using a Gabriel neighboring graph which models the spatial structure of the breeds (Figure S5).

As detailed in Table S5, the first sPCA eigenvalue was strikingly large compared to the others and similar in magnitude to the first PCA eigenvalue. In addition, the genetic variance associated to the first PCA component was found only slightly higher (13% of the total variance) than the corresponding sPCA one (12% of the total variance). Correspondingly, spatial autocorrelation on the first axis, as measured by the Moran's *I* [29] was high in both analyses (*I_1_^PCA^* = 0.73 and *I_1_^sPCA^* = 0.87). Hence the first axis in both PCA and sPCA unambiguously captures global spatial patterns while separating populations according to a North/South gradient (Figure S6A). Nevertheless, on subsequent PCA axes, spatial autocorrelation appeared very low (*I_2_^PCA^* = 0.14 and *I_3_^PCA^* = 0.06) while the second and third sPCA axes displayed a Moran's *I* above 0.5 (*I_2_^sPCA^* = 0.63 and *I_3_^sPCA^* = 0.80). This suggested that PCA might fail to identify relevant spatial patterns on these additional axes making it difficult to interpret the underlying variance in terms of geography. We thus focused subsequently on the first three sPCA axes (Figure S6). Note that some axes, such as axis 22 (*I_22_^sPCA^* = −0.63) displayed a relatively high negative spatial autocorrelation suggesting a strong local spatial pattern. This axis actually separated JE2 and GNS (data not shown) which are closely geographically related (Channel Islands) but most probably because of complete isolation of JER since the 18^th^ century [2] are clearly genetically distinct (see above).

**Figure 5:**
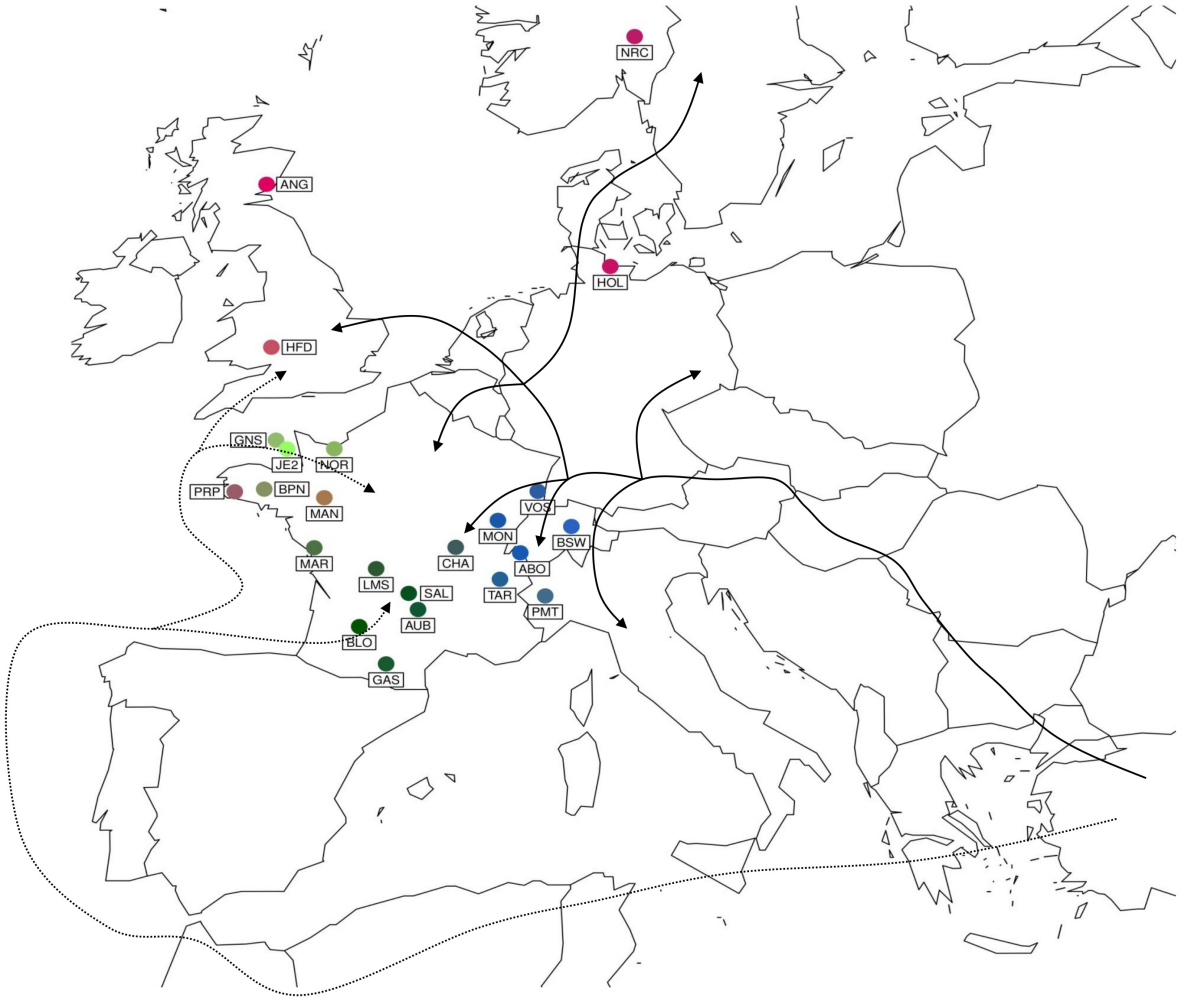
Projection on a map of Europe of the colorplots synthesizing the breed coordinates on the three first sPCA principal components. These plots can show up to three coordinates at the same time by translating each coordinate into a channel of color (Red, Green, and Blue). The obtained values are used to compose a color under the RGB system. The Danubian (solid line) and Mediterranean (dashed line) migration routes are also reproduced on the map [3].

The coordinates of each breed on the first three sPCA axes were synthesized on Figure 5 by means of colorplots [30], [31] projected on the geographic map. Based on the different colors obtained (see also Figure S6D for a 3D representation), four groups of breeds showed high geographical consistency in good agreement with the NJ tree results (Figure 4). The underlying four groups of colors were i) the dark green one which comprises 5 breeds (LMS, SAL, AUB, BLO and GAS) from central and southwestern France, ii) the light green one which comprises 4 breeds (JE2, GNS, NOR and BPN) from the Channel Islands and northwestern France iii) the blue one which comprises 6 breeds (VOS, MON, ABO, TAR, BSW, and PMT) from Eastern France and the Alps and iv) the brown red one which comprises 6 breeds (ANG, NRC, HFD, HOL, PRP, MAN) from Northern Europe origin. However, CHA and MAR remained difficult to assign to one of these four groups owing to their low scores on the first three sPCs.

The grouping of French breeds is mostly in agreement with previous classification based on historical data, morphological characters (mostly craniometric and morphometric data), geographical proximity [32] and blood groups, transferrin and β-casein polymorphisms [33]. In addition, they are quite consistent geographically. Two notable exceptions are represented by the PRP and MAN which belong to the North European breed group (iv) although originating from an area closer to i) and ii) confirming results from a previous study based on microsatellite markers [34]. These inconsistencies between genetic and geographical data are actually expected since the PRP has been recently derived from the red Holstein and the Meuse-Rhin-Yssel breed from Germany. Similarly, introgression of British Durham during the 19^th^ century had been extensively reported in MAN. The grouping of the NOR with the Channel Islands and Northwestern France breeds (ii) used to be more controversial [33] although in agreement with early classification based on biochemical markers [35]. Finally, it should be noted that in our study the position of CHA differed according to the methods used. Consistently, some historical data established a connection between CHA and the South-Western France blonde breeds as in the tree of Figure 4 [33] while the most commonly accepted theory used to associate CHA with the Jurassic group (represented in our study by MON and VOS in our study) [32]. Overall, sPCA results might be related to archeological and historical data [3]. Indeed the geographical positions of the four identified groups are in agreement with the early postulated migration routes by which the Neolithic culture expanded towards France (see Introduction and [3]). Hence, our groups iii) and iv) appeared closely related to the Danubian colonization route while groups i) and ii) might correspond to the Mediterranean colonization route (Figure 5). [2], [4], [10]. However, adding more European populations to our combined data set remains of paramount importance to further demonstrate the influence of the postulated migration routes on the structure of French cattle populations.

More generally, including data for populations from other parts of the world (*e.g.* Southern Europe, Northern Africa, India or Middle East) may provide additional useful insights to draw a more precise picture of the genetic history of cattle. As exemplified in the present study, such extension is straightforward because of the easy to share nature of SNP data and the widespread use and cost effectiveness of the Bovine SNP50 genotyping assay.

## Materials and Methods

### Ethics statement

No ethics statement was required for the collection of DNA samples. DNA was extracted either from commercial AI bull semen straws or from blood samples obtained from different veterinary practitioners visiting farms with the permission of the owners.

### Genotyping data, quality control, marker selection and estimation of LD

For the purpose of this study 296 individuals belonging to 14 different French cattle breeds were genotyped on the Illumina BovineSNP50 chip assay [14] at the Centre National de Génotypage (CNG) platform (Evry, France) using standard procedures (http://www.illumina.com). Based on available pedigree information, every attempt was made to ensure that samples were typical of the breeds and to limit relationships. In addition, genotyping data were also included for 33 other breeds and 4 american bisons (*Bison bison*) [14]–[16]. For these latter studies and for the sake of homogeneity in sample size across the whole data set, the maximal number of individuals retained per breed was restricted to 31 individuals (trying to limit their relationships when pedigree information was available). In total, 1,121 individuals (from 14 to 31 per breed) were available for the different analyses (Table S1).

Description of the origin of samples and genotyping data is detailed in Table 1. Among the 51,582 genotyped SNPs mapping to a bovine autosome on the Btau_4.0 bovine genome assembly [36], 3,009 SNPs which were not genotyped for at least 75% of the individuals in at least one breed and 2,982 SNPs which were monomorphic in all breeds were discarded from further analyses. Notice that all the individuals were genotyped for at least 95% of the selected SNPs. Following [16], an exact test for Hardy-Weinberg Equilibrium (HWE) [37] was further carried out within each breed separately on the 45,291 remaining SNPs. Based on the obtained p-values, q-values [38] were estimated for each SNP using the R package qvalue (http://cran.r-project.org/web/packages/qvalue/index.html). A total of 585 SNPs exhibiting q-value<0.05 in at least one breed were then discarded from further analysis. Thus, 44,706 SNPs were finally considered for the study leading to an average marker density of 1 SNP every 56.9 kb over the genome (Table S2). Moreover, as shown in Figure S2 and detailed in Table S1, the genome coverage was homogeneous with a median distance between consecutive SNPs equal to 40.4 kb. Few large gaps between SNPs were present since the 95th (99th) percentile of this distance was 146 kb (252 kb), the largest gap localized on BTA10 being 2 Mb long. Conversely, less than 0.5% of the distances between successive SNPs were shorter than 20 kb. In order to characterize the extent of LD, we computed the *r^2^* measure [39] between each marker pair within each breed separetely using Haploview 4.1 [40].

### Principal Component Analysis

*PCA* was carried out based on all available SNP information using the R packages *ade4* [41]. Note that, as expected from the extent of LD, multivariate analyses using the SMARTPCA software package [42] which allows to perform correction for the extent of LD (by replacing individual SNP values with the residuals from a multivariate regression without intercept on the two preceding SNPs on the map, provided they are less than 200 kb apart) lead to almost identical results.

### Spatial Principal Component Analysis

sPCA was carried out on a between-breed level using the R packages *ade4* [41] and *adegenet* [30]. Briefly, while in PCA, the optimization criterion only deals with genetic variance (with the eigenvalue decomposition of **X'X**, where **X** is the matrix of allelic frequencies), sPCA aims at finding independent synthetic variables that maximize the product of the genetic variance and spatial autocorrelation measured by Moran's *I* [29]. This is accomplished by the eigenvalue decomposition of a matrix **X'(L+L')X** where L synthesizes spatial structure among populations via a neighboring graph (in our study a Gabriel neighboring graph was chosen) connecting the populations on the geographical map [17], [43] to model spatial structure among breeds. Resulting eigenvalues can be either positive or negative reflecting respectively global or local spatial pattern. Finally, the overall spatial autocorrelation associated to each resulting sPCA principal component was quantified using the Moran's *I* [29]. For a thorough description of sPCA, interested reader should refer to [17].

### Neighbor-Joining trees construction

ASD were computed for each pair of individuals using all available SNP information by a simple counting algorithm: for a given pair of individuals i and j, ASD was defined as 1-*x_ij_* where *x_ij_* represents the proportion of alleles alike in state averaged over all genotyped SNPs. A neighbor-joining tree [44] was computed based on the resulting distance matrix using the R package APE [45]. Similarly a neighbor-joining tree based on the Reynolds genetic distances [46] between the different pairs of breeds was constructed using PHYLIP 3.65 package [47]. The reliability of each node was estimated from 100 random bootstrap resamplings of the data. The resulting dendrogram in Figure 4 was plotted using the program Dendroscope [48].

### Unsupervised Hierarchical Clustering of the individuals

Unsupervised hierarchical clustering of individuals based on SNP genotyping data was performed using the maximum likelihood method described in [21] which is implemented via an Expectation-Maximization algorithm in the program *frappe*. The program was allowed to run for 10,000 iterations, with pre-specified numbers of clusters varying from K = 2 to K = 47 (number of distinct populations). Convergence of the algorithm was empirically assessed by considering estimated cluster membership and data likelihood. Graphical displays of the results were done with the program *Distruct* [49].

### F-statistics

The global *F-*statistics *F_IT_*, *F_ST_* and *F_IS_* were estimated respectively in the form of *F*, *θ* and *f* [50] using the program GENEPOP 4.0 [51]. GENEPOP 4.0 was also used to estimate diversity for each locus and population both within individuals and among individuals within a population. The within breed *F_IS_* was derived from the average of these two quantities over all the SNPs. In order to evaluate the reliability of the *F_IS_* estimates we computed the mean and standard deviation over 10,000 samples of 5,000 randomly chosen SNPs.

## Supporting Information

Figure S1 Decay of average pairwise r2 with inter-marker distance for the different populations.
(0.05 MB PDF)

Figure S2 Distribution of inter-SNP physical distances based on the Btau_4.0 bovine genome assembly (http://genome.ucsc.edu/).
(0.00 MB PDF)

Figure S3 Between breed PCA for the 47 different bovine populations. Populations are plotted according to their coordinates on the first two (A) and first and third (B) principal components on the eigenanalysis.
(0.12 MB PDF)

Figure S4 Neighbor-Joining tree relating the 47 cattle populations and American bisons (OBB) outgroup based on Reynolds genetic distances computed using allele frequencies at 27,527 SNPs polymorphic (MAF>0.01) in at least two zebus, two WAT and two EUR breeds. Reliability of the nodes (percentage over 100 Bootstrap samples) are indicated for each node.
(0.05 MB PDF)

Figure S5 Gabriel neighboring graph modeling the spatial structure of breeds projected on the geographic map.
(0.03 MB PDF)

Figure S6 sPCA results. Projection of the breed coordinates on the first (A), second (B) and third (C) sPCA principal components onto the geographical map. The area of the square is proportional to the absolute value of the score while the color of the square (black or white) corresponds to its sign (positive or negative). D) 3D representation of the breed coordinates on the first three sPCA principal components (breed names are colored according to the synthetic score obtained in Figure 4 representation).
(0.10 MB PDF)

Table S1 Sample Description. The land of origin, country of sampling (N = North, S = South, SE = South East, SW = South West, NE = North East, NW = North West and M = Middle) and type of the populations (EUT = European Taurines, SYN = Synthetic breeds, NAT = North African Taurines, WAT = West African Taurines, WAH = West African Hybrids, WAZ = West African Zebus, EAZ = East African Taurines and ZEB = Zebus from Indian origin) are indicated, together with the number of individuals sampled. For each breed, marker polymorphism is summarized through average heterozygosity computed across the 44,706 SNPs considered in this study and the proportion of SNPs with a MAF above 0.05.
(0.03 MB XLS)

Table S2 SNP bovine genome coverage based on the Btau_4.0 bovine genome assembly (http://genome.ucsc.edu/).
(0.01 MB XLS)

Table S3 Within population F_IS_.
(0.01 MB XLS)

Table S4 F_ST_ for each pair of populations.
(0.05 MB XLS)

Table S5 Comparison of PCA and sPCA. For each analysis, eigenvalues, percentage of genetic variance explained and Moran's *I* spatial autocorrelation associated to the corresponding principal components are given.
(0.01 MB XLS)
