# Supplementary figures and images for: Insights into the Genetic History of French Cattle from Dense SNP Data on 47 Worldwide Breeds
Source: PLoS One. 2010 Sep 30;5(9):e13038. doi: 10.1371/journal.pone.0013038 (PMC2948016; doi:10.1371/journal.pone.0013038)

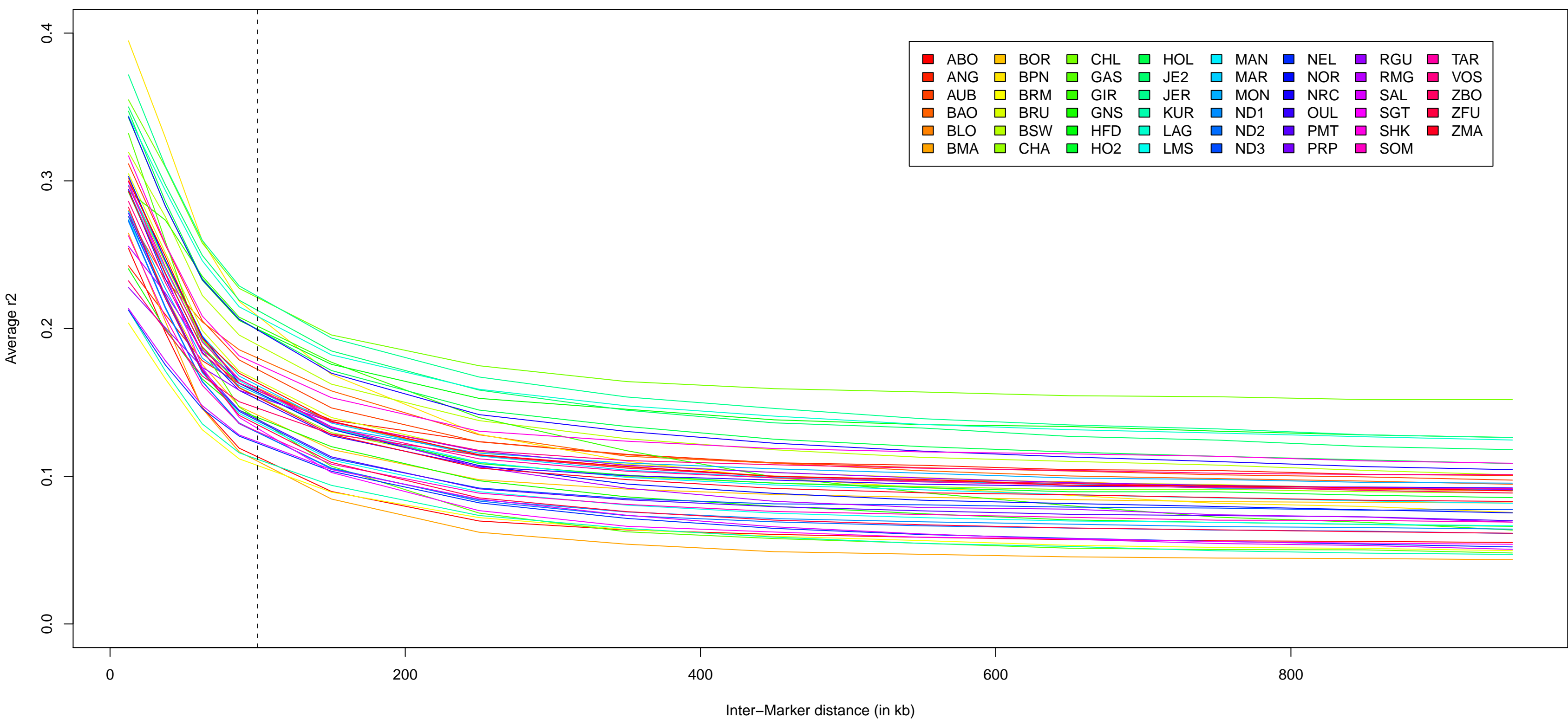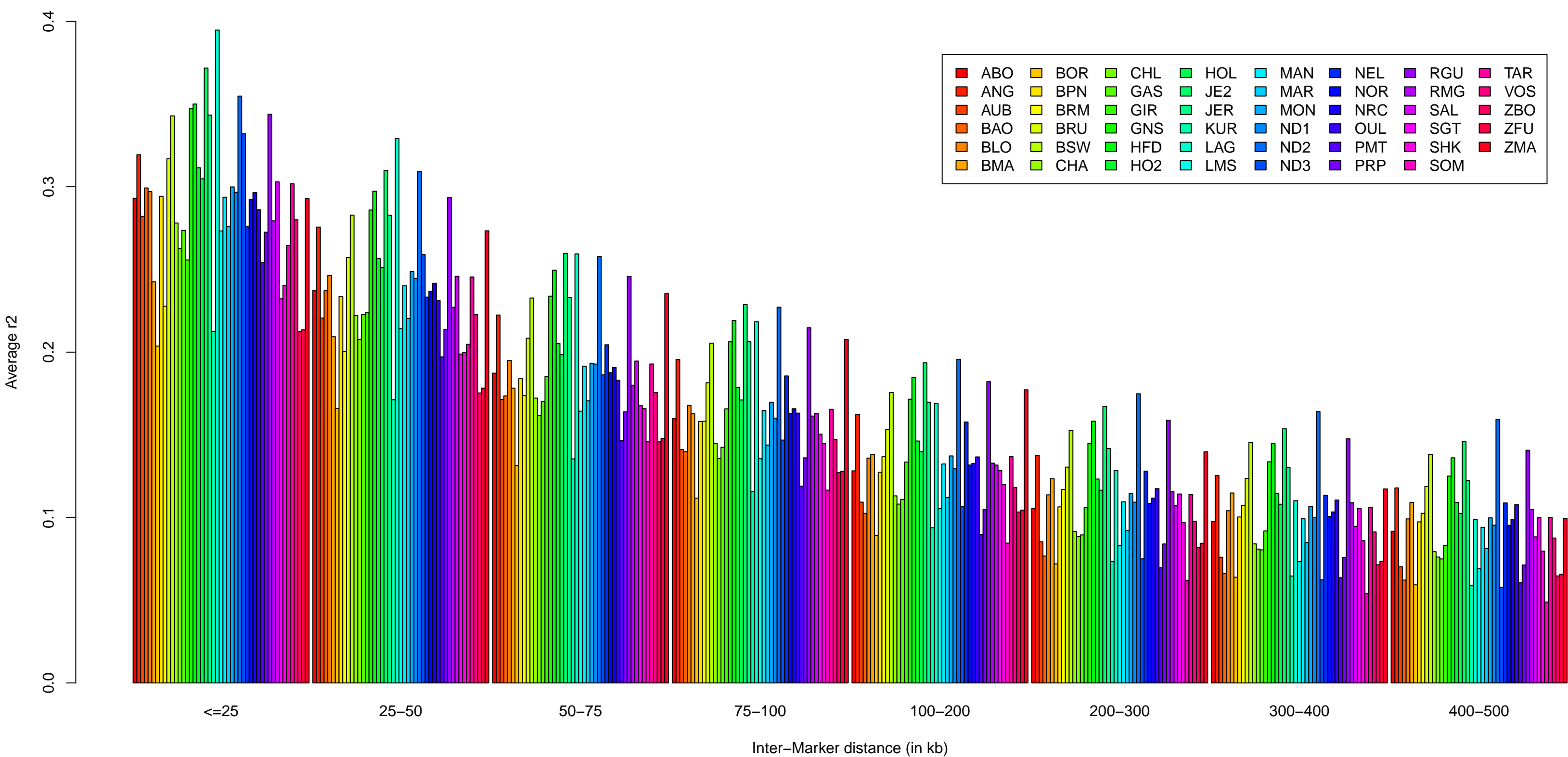

Supplement: Figure S1 — Decay of average pairwise r2 with inter-marker distance for the different populations. (0.05 MB PDF) [file pone.0013038.s001.pdf]

**Distribution of distances between successive SNPs**

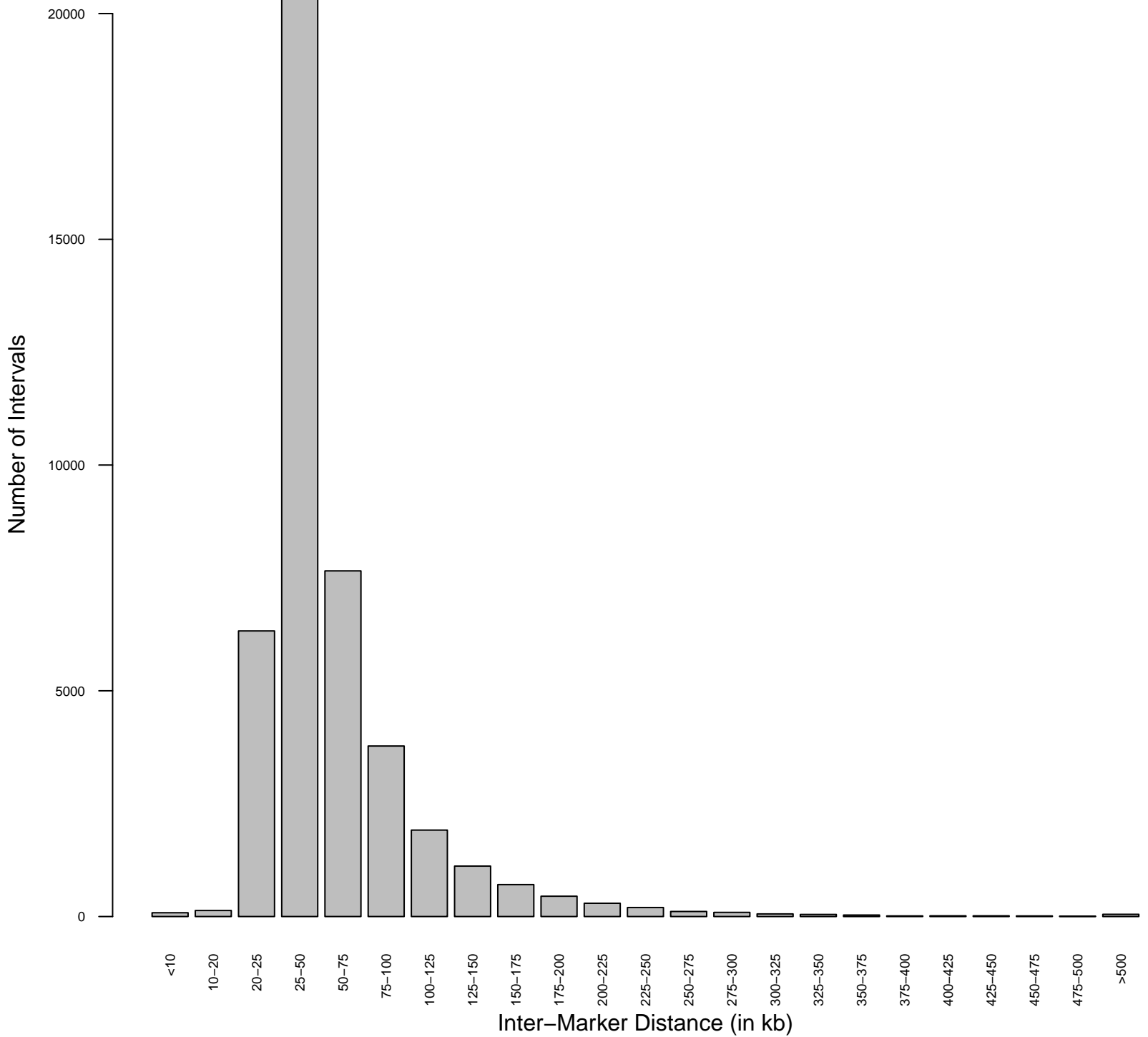

Supplement: Figure S2 — Distribution of inter-SNP physical distances based on the Btau_4.0 bovine genome assembly (http://genome.ucsc.edu/). (0.00 MB PDF) [file pone.0013038.s002.pdf]

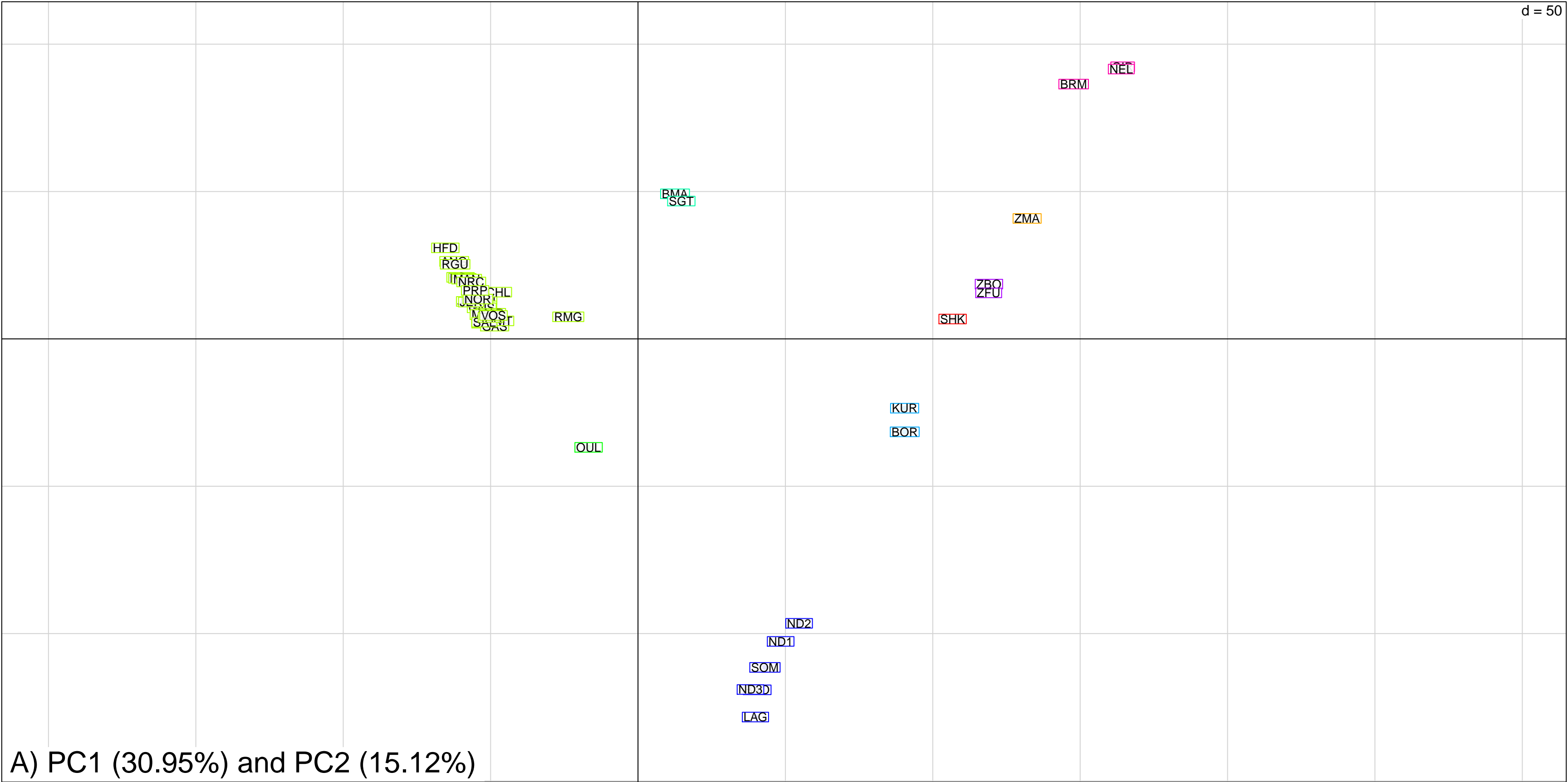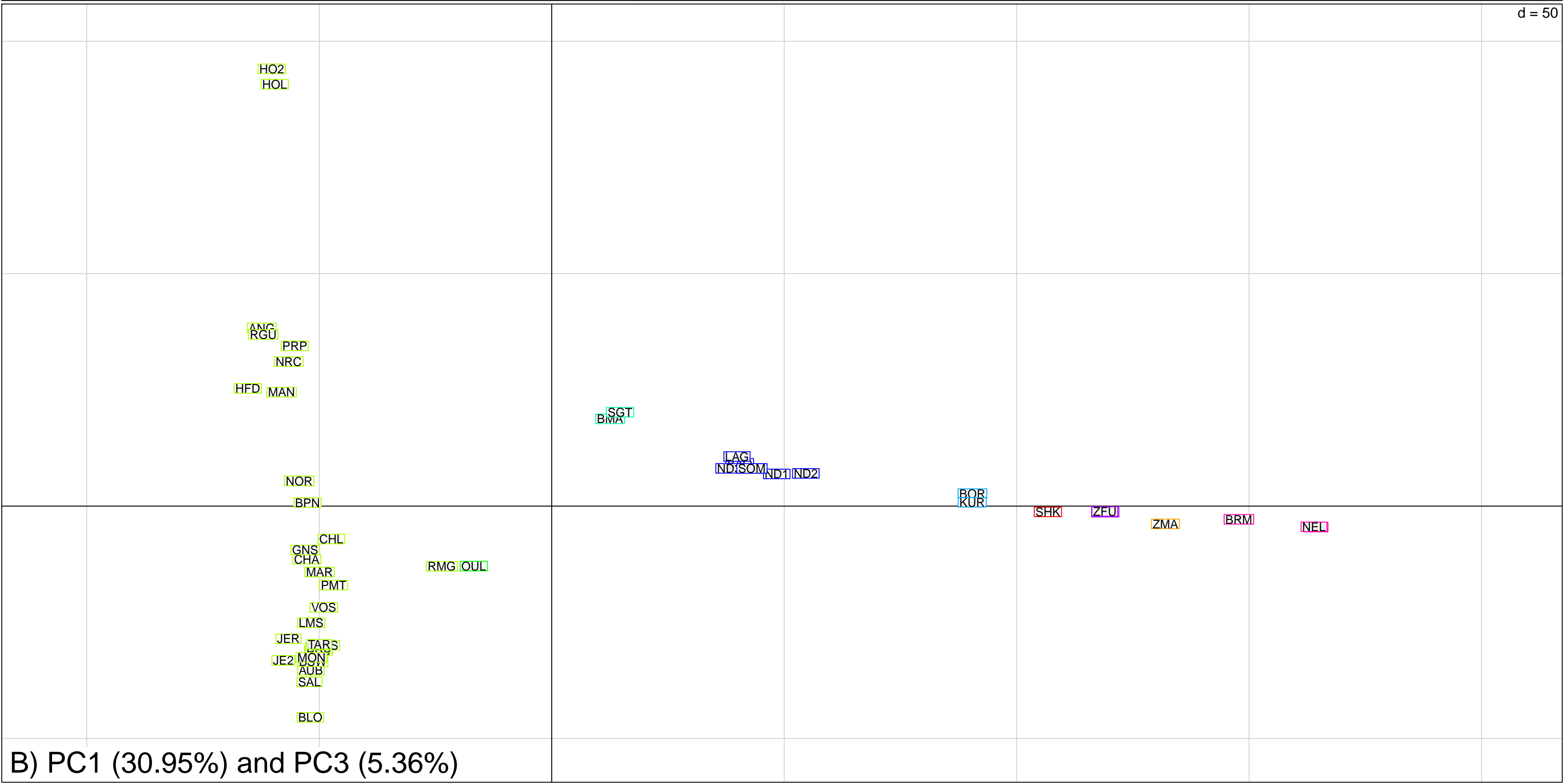

Supplement: Figure S3 — Between breed PCA for the 47 different bovine populations. Populations are plotted according to their coordinates on the first two (A) and first and third (B) principal components on the eigenanalysis. (0.12 MB PDF) [file pone.0013038.s003.pdf]

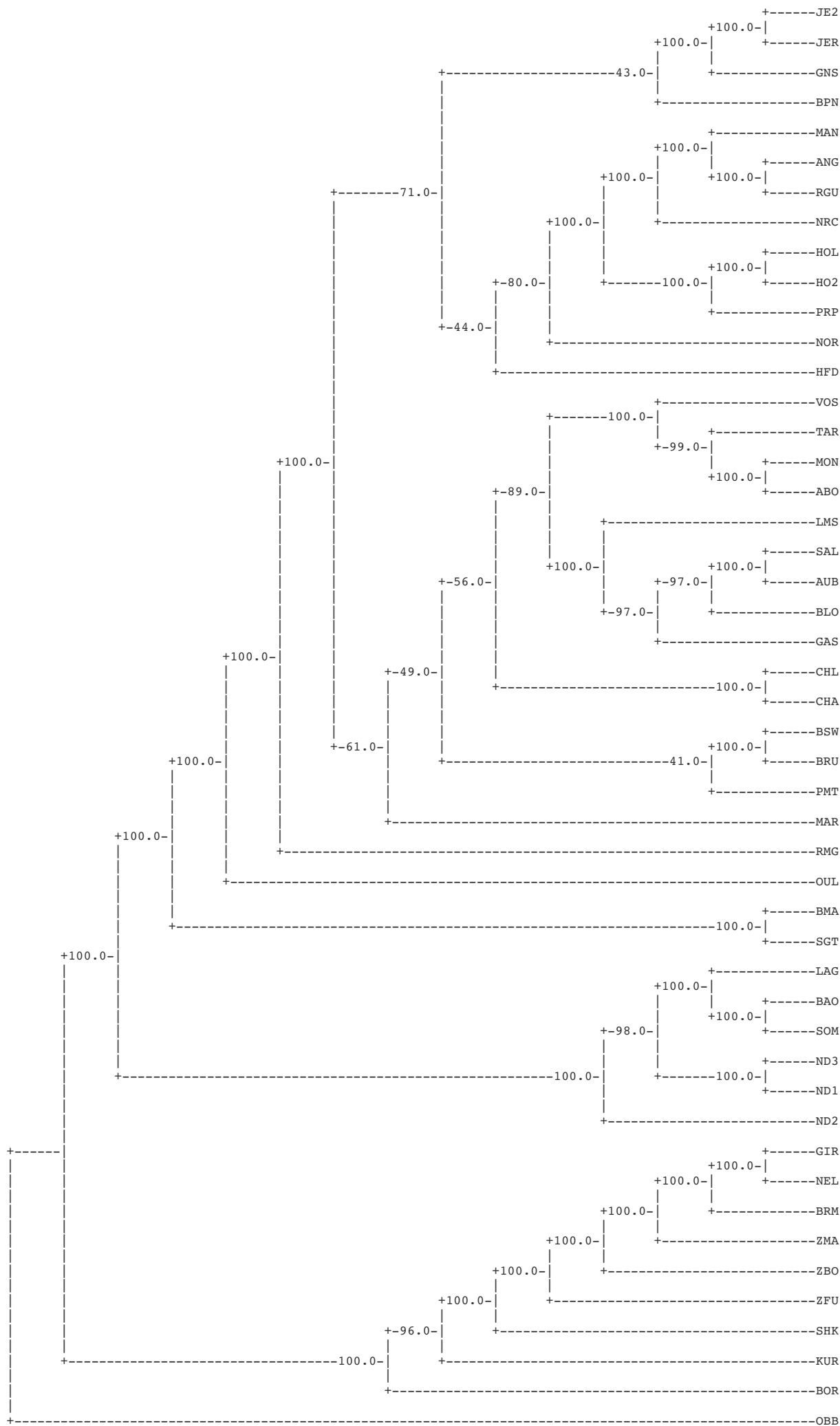

Supplement: Figure S4 — Neighbor-Joining tree relating the 47 cattle populations and American bisons (OBB) outgroup based on Reynolds genetic distances computed using allele frequencies at 27,527 SNPs polymorphic (MAF>0.01) in at least two zebus, two WAT and two EUR breeds. Reliability of the nodes (percentage over 100 Bootstrap samples) are indicated for each node. (0.05 MB PDF) [file pone.0013038.s004.pdf]

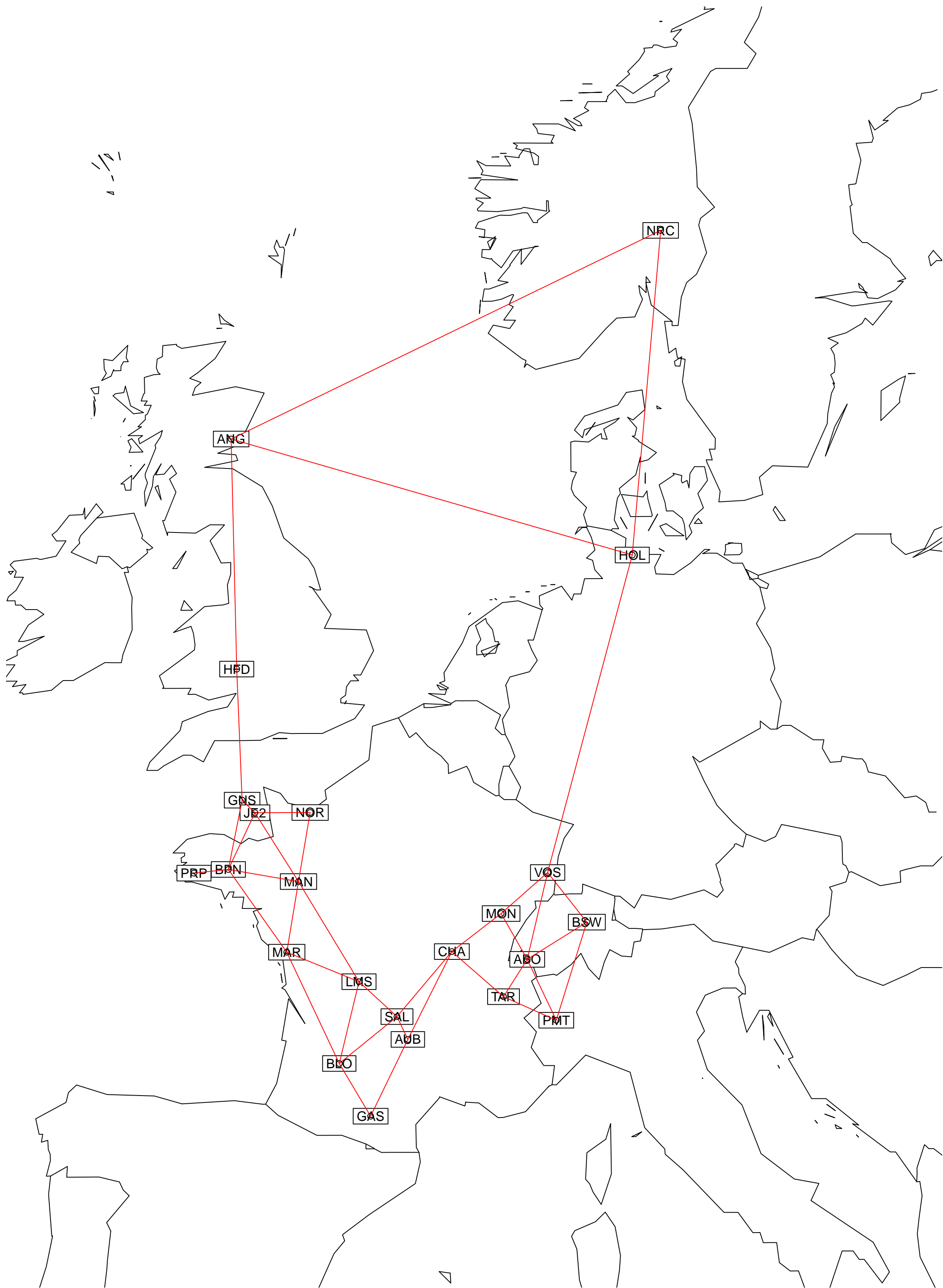

Supplement: Figure S5 — Gabriel neighboring graph modeling the spatial structure of breeds projected on the geographic map. (0.03 MB PDF) [file pone.0013038.s005.pdf]

A) Axis 1

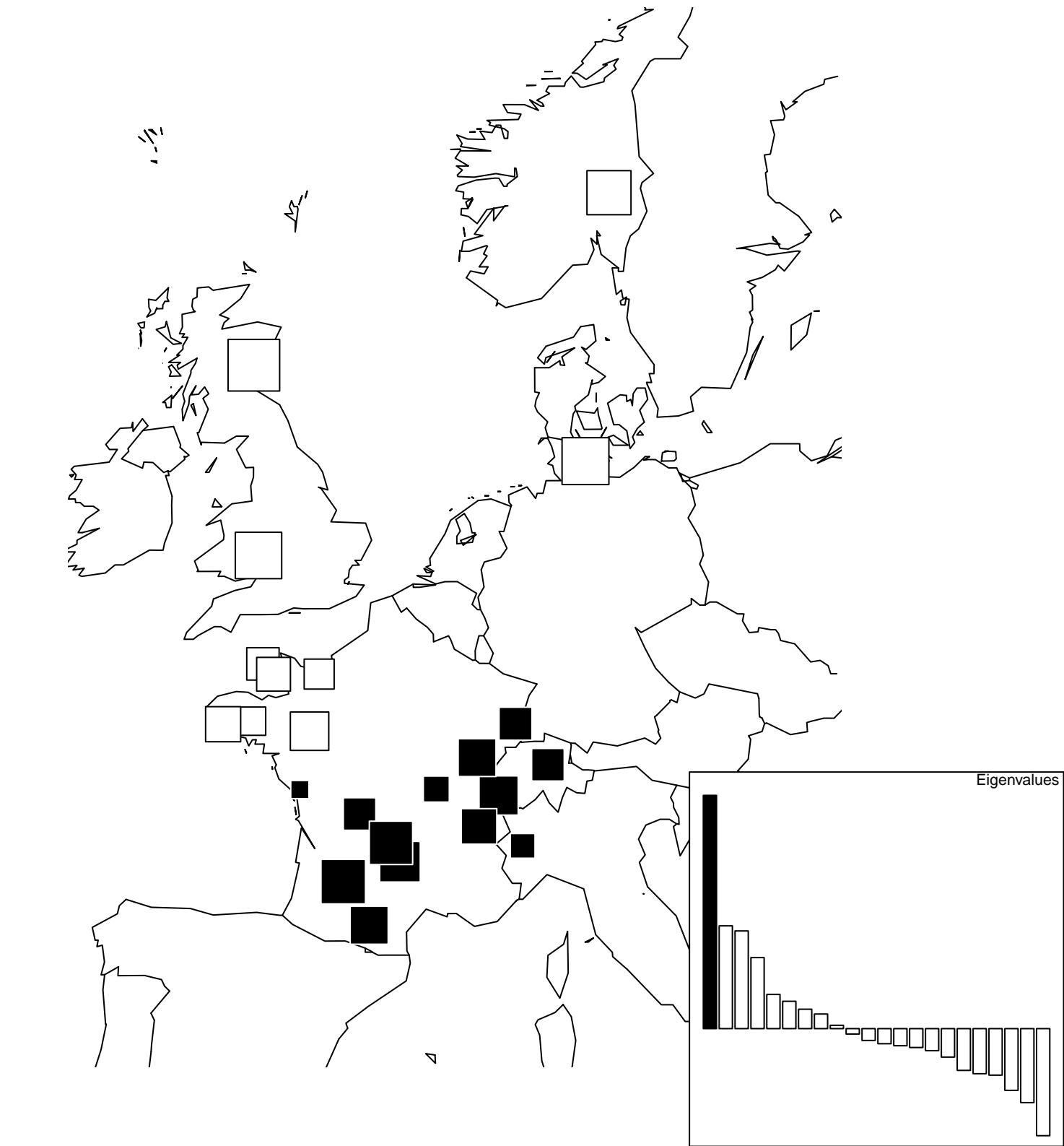

B) Axis 2

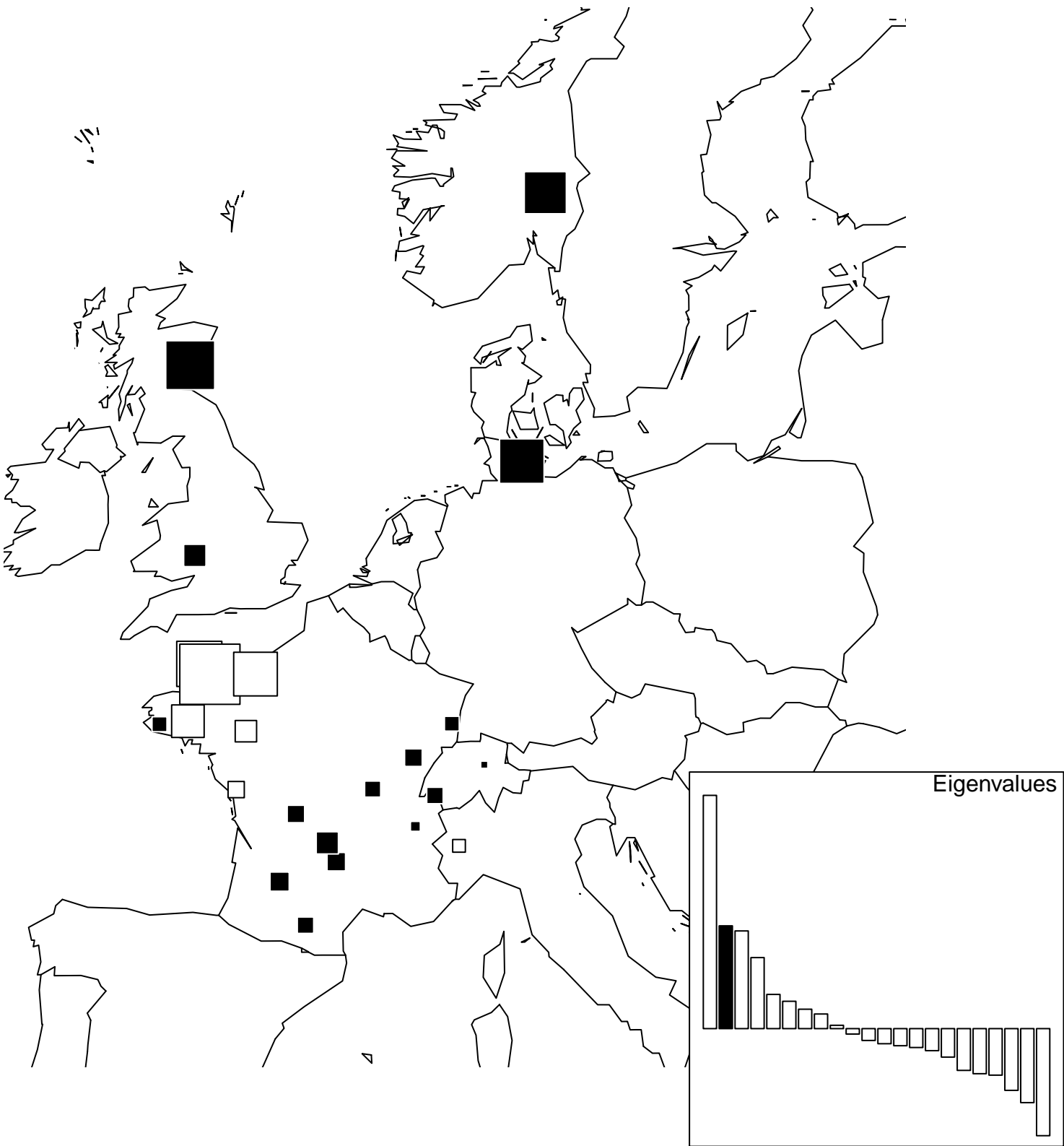

C) Axis 3

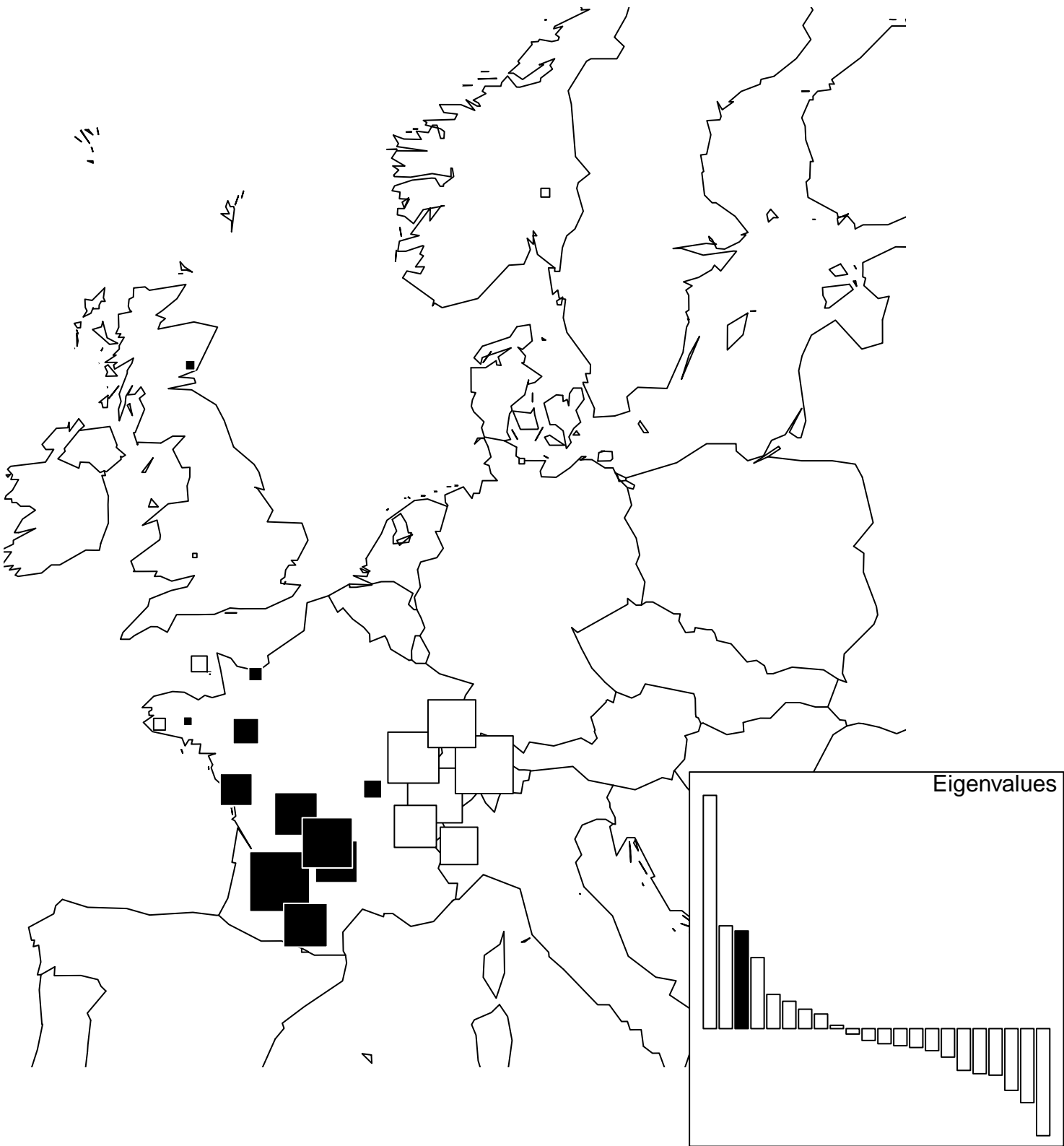

D) Axes 1,2 and 3

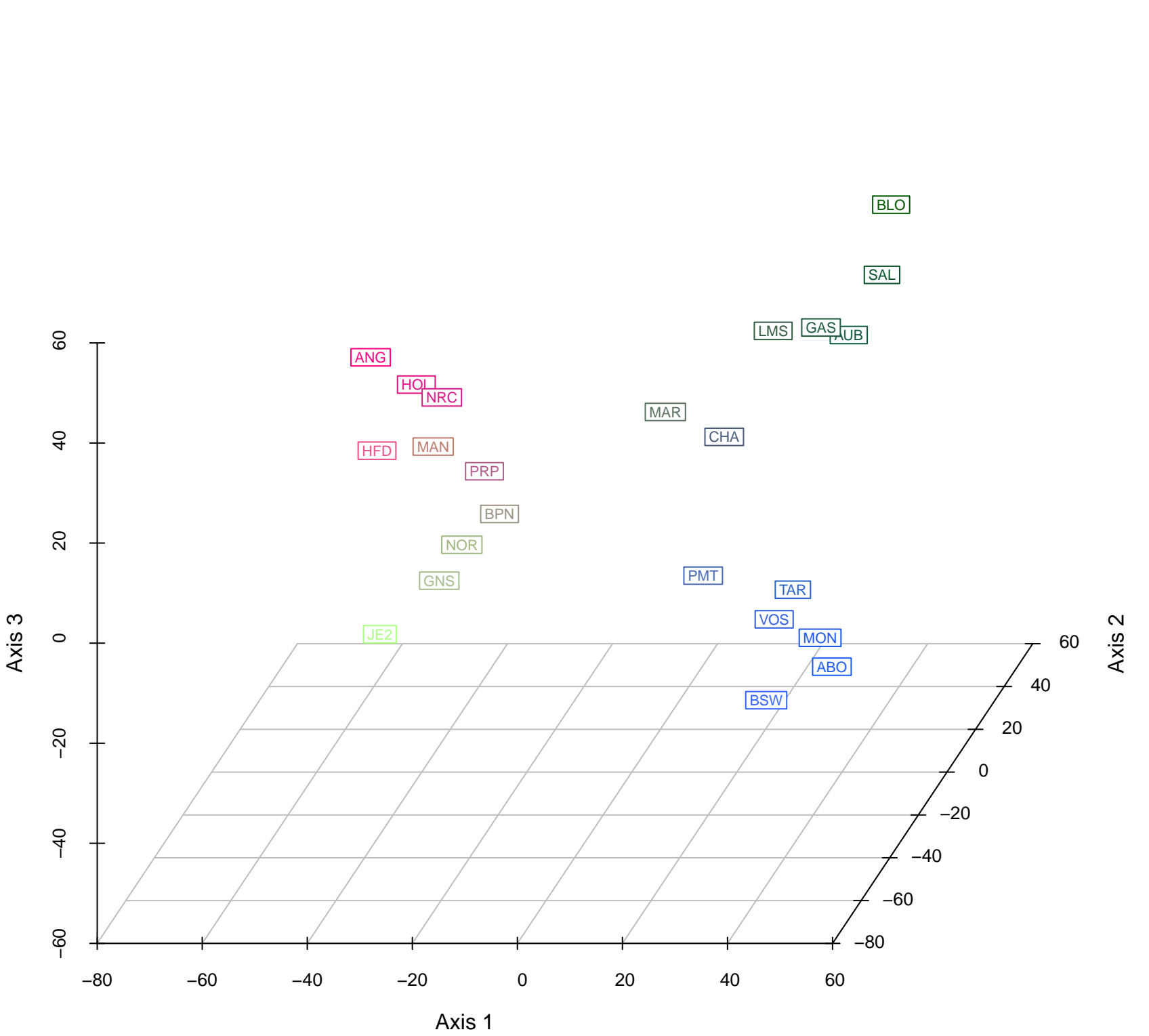

Supplement: Figure S6 — sPCA results. Projection of the breed coordinates on the first (A), second (B) and third (C) sPCA principal components onto the geographical map. The area of the square is proportional to the absolute value of the score while the color of the square (black or white) corresponds to its sign (positive or negative). D) 3D representation of the breed coordinates on the first three sPCA principal components (breed names are colored according to the synthetic score obtained in Figure 4 representation). (0.10 MB PDF) [file pone.0013038.s006.pdf]
